# Chemical and Functional Properties of Chickpea (*Cicer arietinum* L.)-Based Fermented Beverages Produced Using Different Lactic Acid Bacteria

**DOI:** 10.3390/foods15030523

**Published:** 2026-02-03

**Authors:** Angela Pazzanese, Silvia Tagliamonte, Maria Aponte, Giuseppe Blaiotta, Manuela Flavia Chiacchio, Prakriti Khanal, Paola Vitaglione

**Affiliations:** Department of Agricultural Sciences, University of Naples Federico II, 80055 Portici, Italy; angela.pazzanese@unina.it (A.P.); silvia.tagliamonte@unina.it (S.T.); aponte@unina.it (M.A.); manuelaflavia.chiacchio@unina.it (M.F.C.); prakriti.khanal@googlemail.com (P.K.); paola.vitaglione@unina.it (P.V.)

**Keywords:** fermentation, plant-based food, chickpea, beverage, antioxidant activity, in vitro digestion, total polyphenol content

## Abstract

Fermentation can enhance the technological properties and nutritional value of legumes. This study aimed to develop an innovative chickpea-based fermented beverage with yeast in combination with lactic acid bacteria (LAB) strains. Autochthonous cultures isolated from chickpea soaking water, along with LAB strains from previous studies, were used to produce fermented chickpea beverages. Hydrolyzed chickpea flour was inoculated with LAB (*Lactiplantibacillus*, *Lacticaseibacillus*, *Lentilactobacillus*, *Leuconostoc*, *Pediococcus*, and *Weissella*) and 2 yeast (*Metschnikowia* and *Saccharomyces*) strains. Growth performance, phytic acid content, and total antioxidant capacity (TAC) were evaluated. In a second phase, four fermented beverages were produced by co-fermenting Saccharomyces cerevisiae with the four best-performing LAB strains. Microbial growth and pH were monitored throughout fermentation, and beverages were analyzed for TAC and Total Polyphenol Content (TPC) before and after in vitro digestion. The beverages exhibited high microbial viability and increased TAC and TPC compared to controls. Although both parameters decreased after in vitro digestion, their values remained higher than those of the controls. The combination *Saccharomyces cerevisiae* LN7/*Lactiplantibacillus plantarum* 95 proved to be the most effective. Results highlight the importance of the strains selection in enhancing the antioxidant properties and polyphenol content of plant-based fermented beverages and provide insight into the effects of digestion on their functional properties.

## 1. Introduction

The promotion of human health and well-being through sustainable and healthy dietary patterns is a central objective of the United Nations’ Agenda 2030 [1]. In this context, the agri-food sector is increasingly encouraged to develop alternatives to the Western dietary pattern, which is characterized by high consumption of processed red meat and refined sugars and is associated with negative health and environmental outcomes. Diets rich in fruits, vegetables, legumes, nuts, seeds, and whole grains are widely recognized as more sustainable and health-promoting options [2].

Legumes represent an affordable, nutritionally dense, and environmentally sustainable alternative to animal-derived foods [3]. Among them, chickpea (*Cicer arietinum* L.) is particularly attractive due to its high content of plant protein, essential amino acids, dietary fiber, unsaturated fatty acids, vitamins, and minerals. Chickpeas are naturally free of lactose and cholesterol and contain carbohydrates with a low glycemic index [4,5]. In addition, chickpeas are a source of bioactive compounds including phytochemicals (i.e., phenolic acids, flavonoids) and small bioactive peptides (BAPs), which have been associated with antioxidant, anti-inflammatory, and antihypertensive activities as well as cholesterol- and glycemia-lowering effects [6].

Despite these nutritional and functional attributes, legume consumption remains below recommended levels in many regions [7]. This is partly due to the presence of antinutritional factors such as raffinose-family oligosaccharides, phytates, tannins, and saponins [8], which can negatively affect digestibility and consumer acceptance, as well as to long preparation times that are incompatible with modern dietary habits [9]. Fermentation by lactic acid bacteria (LAB) has emerged as an effective and sustainable strategy to improve the nutritional quality and functionality of legume-based matrices. LAB fermentation has been shown to reduce anti-nutritional factors and oligosaccharides, enhance protein digestibility and free amino acid content, increase antioxidant activity and total polyphenol content, and promote the formation of BAPs [10,11,12,13,14]. However, these effects are highly strain-dependent, and the selection of appropriate microbial cultures remains a key challenge in the development of fermented legume-based foods [10].

Fermented legume-based beverages, in particular, represent a promising approach to increasing legume intake by providing convenient, ready-to-consume products that are gluten-free, lactose-free, cholesterol-free, and may contain viable microorganisms [14,15]. Although several plant matrices have been explored for fermented beverage production, chickpeas and faba beans remain relatively underexploited by the food industry, especially in comparison with soy, oat, or almond-based products [16]. Moreover, limited information is available on the combined use of LAB and yeast strains, including autochthonous microorganisms, for the fermentation of chickpea-based beverages and on their impact on antioxidant properties and bioaccessibility after digestion.

Within this framework, the present study aimed to address this knowledge gap by developing and characterizing a chickpea-based fermented beverage obtained through co-fermentation with LAB and yeast. Specifically, the aims of the study were as follows: (i) to evaluate the technological performance of LAB and yeast strains isolated from the chickpea ecosystem and from other food environments during the fermentation of chickpea hydrolysates; (ii) to assess the effect of microbial selection and fermentation on the chemical and functional properties of the beverages, with particular emphasis on total antioxidant capacity (TAC) and total polyphenol content (TPC); and (iii) to investigate the antioxidant activity and polyphenol bioaccessibility of the fermented beverages using an in vitro digestion model.

## 2. Materials and Methods

### 2.1. LAB Isolation from Chickpea Seeds

Chickpea (*Cicer arietinum* L.) seeds of the Cicerale and Caposele cultivars were purchased by local producers. Cicerale is an ancient cultivar typical of the homonymous town in the Salerno Province (Campania Region, Southern Italy) and is a Slow Food Presidia (https://www.fondazioneslowfood.com/en/slow-food-presidia/cicerale-chickpea/ (accessed on 1 September 2025). Similarly, the cultivar Caposele is an old cultivar produced in the town by the same name in the regional Park of Monti Picentini in the Avellino Province (Campania). Specifically, two samples of Cicerale (CIC1 and CIC2) and one sample of Caposele (CAP) were analyzed. Whole chickpea seeds of each sample were selected, weighed, and divided into three batches of 50 g each. Each batch was placed in a sterile 250 mL Erlenmeyer flask and mixed with 2.5 volumes of sterile Milli-Q water. After soaking for 16 h at 25 °C, legumes were taken off, and the water was incubated for a further 24 h (T40). Soaking water was analyzed at time 0 (T0 h) and after 16 (T16 h) and 40 (T40 h) hours. pH was monitored by using a pH60 VioLab pH-meter XS (Giorgio Bormac S.r.l., Modena, Italy). Reducing sugars and organic acids were evaluated by High-Performance Liquid Chromatography (HPLC) analysis. A Gilson 307 Series HPLC system equipped with a refractive index detector (RID 133, Gilson Inc., Middleton, WI, USA) and a MetCarb68H column (6.5 × 300 mm, Varian Agilent, Santa Clara, CA, USA) was used [17] to analyze 20 mL of soaking water in triplicate. The flow rate was 0.4 mL/min, and the mobile phase was 0.01 N H_2_SO_4_. The temperature of the column was set at 65 °C. Glucose and fructose were identified by comparing their retention times with those of standards under the same HPLC conditions. Quantitative determination was performed using the external standard method.

LAB were counted on MRS agar (Oxoid, Basingstoke, UK) supplemented with cycloheximide (200 mg/L) after incubation at 30 °C for 72 h. Bacterial spores were evaluated on PCA (Oxoid) after heat treatment (80 °C for 15 min), solely in samples T40 h. Plates were incubated in aerobiosis for 48 h at 30 °C. Colonies were randomly isolated from plates seeded with the highest dilutions of samples T16 h and T40 h. After purification, by repetitive streaking, cultures were identified by both MALDI-TOF (Bruker MALDI Biotyper, Bremen, Germany) and/or by 16S rDNA partial sequencing by using primers and PCR conditions described by Weisburg et al. (1991) [18]. DNA was extracted using the NucleoSpin Food kit (Macherey-Naggel GmbH &Co KG, Duren, Germany). Sequencing was carried out by Eurofins Genomics (Ebersberg, Germany).

### 2.2. Chickpea Flour Hydrolysate Production

Chickpea flour from cv. Cicerale, made in a stone mill and sieved at 220 µm, was provided by a local producer. The proximal composition (% *w*/*w*) was moisture 14.97%, total carbohydrates 48.0% (of which sugars, 3.5%), dietary fiber 9%, lipids 6.6%, proteins 21.0%, and salt 0.03%. Chickpea flour was blended with de-ionized water (10% *w*/*v*) and kept in a water bath (Julabo TW8 water bath) at 50 °C, then Alfa-Amylase (1 mL/L) and glucoamylase (475 U/mL, ABV Glucoamylase 400-GAG 511-Lallemand Brewing, Montreal, QC, Canada) (1 mL/L) were added. The used thermostable Alfa-Amylase (3000–4000 U/mL) possesses proteolytic (600–700 U/mL), glucanasic, and cellulolytic (Endozyme Brewmix, AEB SPA, Brescia, Italy) activities as well. After a 30 min incubation, the mixture was heated at 65 °C for 60 min and then at 100 °C for 10 min. During the hydrolysis, soluble solids were refractometrically monitored (°Brix, Portable Refractometer, PBI International, Milan, Italy), while soluble sugars (glucose and fructose) were determined by HPLC analysis at the beginning and the end of the hydrolysis. Samples were diluted 1:5 (*w*/*v*) in ultrapure water, centrifuged at 6000 rpm (rotor A8-50, NEYA-XS, Sinergica Soluzioni, Milan, Italy), and filtered by AcroDisc millipore (0.2 μm). Water extracts (20 mL) were then analyzed by HPLC. The flour mixture devoid of enzyme served as a control.

### 2.3. Microorganisms and Growth Conditions

The source of LAB and yeast strains used in this study is reported in Table 1. Working cultures were prepared in YPD (yeast extract 10 g/L, peptone 20 g/L, dextrose 10 g/L) or MRS for yeast and LAB, respectively. Strains were stored at −20 °C in the same two media, added with sterile glycerol (25% *v*/*v*).

### 2.4. Cultures’ Characterization

LAB strains to be used in fermentation were evaluated for biochemical features of technological interest. Fructose, maltose, arabinose, saccharose, and maltodextrin fermentation was tested as described by Moschetti et al. [22]. Urease activity and production of biogenic amines (tyramine and histamine) were evaluated according to Mora et al. [23] and Bover-Cid and Holzapfel [24], respectively. Starch hydrolysis was assessed on drop-inoculated starch agar plates as described by Blaiotta et al. [25]. Exopolysaccharides (EPS) production was tested on both modified MRS (MRS supplemented with 100 g/L of sucrose) and on modified dextransucrase-inducing agar [26].

Activity against molds was first assessed using the overlay method, as described by Magnusson and Schnurer [27], against a *Penicillium* (*P*.) *roqueforti* strain. After LAB inoculation (two 2 cm-long lines), MRS agar plates were incubated at 30 °C for 48 h in anaerobic jars and then overlaid with 10 mL of Malt Extract soft agar containing 10^4^ conidia collected from Malt Extract agar after a seven-day incubation at 25 °C. After 48 h at 30 °C, plates were examined for clear zones of inhibition around the bacterial streaks. Results were confirmed by the Mold agar spot assay described by Schillinger and Villarreal [28] and modified according to Blaiotta et al. [29] by using MRS and MRS devoid of acetate for the LAB growth. Briefly, LAB filter-sterilized culture supernatants were mixed with Malt Extract agar (Agar Low EEO for Molecular Biology, Microtech, Italy) at 55 °C and poured into plates. Ten μL of the *P. roqueforti* spore (10^6^) suspensions were dropped on the agar, and the plates were incubated for 4 days at 30 °C. The diameter of the mold colonies was measured and compared with the controls: supernatant replaced by MRS or MRS with pH lowered to 4 with HCL 5 M and, alternatively, with DL-lactate.

The antimicrobial activity against bacilli was carried out by using strain *Shouchella* (*Shou*.) *clausii* (formerly *Bacillus clausii*) UBBC 07 (Unique Biotech Ltd., Hyderabad, India) as an indicator. After LAB spot inoculation, MRS agar plates were incubated at 30 °C for 24 h and then covered with 10 mL of Luria–Bertani soft agar seeded with the indicator. Inhibition was scored by measuring the inhibition halos surrounding spots after a further 24 h of incubation at 37 °C. Bactericidal activity was also assessed against *Enterococcus* (*Ent.) hirae* ATCC 10,551 (ex DSM3320), *Staphylococcus* (*Staph.*) *aureus* DSM 939 and *Escherichia* (*E.*) *coli* DSM 799 by loading 50 µL of cultures in exponential phase of growth into wells realized on TSA (*E. coli* and *Staph. aureus*) or MRS (*Ent. hirae*) agar plates seeded with indicators (500 µL). Tests were all conducted in duplicate.

### 2.5. Fermentation of Chickpea Flour Hydrolysates

#### 2.5.1. Cultures’ Fermentation Abilities

Fermentation performances of strains listed in Table 1 were evaluated after incubation at 30 °C for 24 h. Overnight cultures in MRS or YPD, for LAB and yeast, respectively, were used as inoculum. In the fermentation of chickpea flour hydrolysates, the initial population level was fixed at 7 and 6 Log CFU/mL for bacteria and yeast, respectively. Fermentations were carried out in triplicate at 30 °C for 48 h. pH was measured after microbial inoculation (T0) as well as after 24 (T24) and 48 (T48) hours using a pH-meter (XS, model pH 50). For microbial counts, samples were serially diluted in sterile saline solution (NaCl 8.5 g/L, peptone 1.0 g/L, tween 80 0.5 g/L). LAB were counted on MRS agar containing 200 mg/L of cycloheximide. Yeasts were counted on WL nutrient agar with chloramphenicol (100 mg/L). Incubations were carried out at 30 °C for 48–72 h. HPLC analysis for metabolite detection/quantification was performed on the sample water extracts prepared as described in Section 2.1. All samples were freeze-dried by Heto LyoLab 3000 (Fisher Scientific, Loughborough, UK), cooled, and stored in a dry place before the assessment of the Total Antioxidant Capacity (TAC) and the phytic acid content.

#### 2.5.2. Phytic Acid Content

The content of the antinutrient phytic acid was determined using the Phytic Acid Assay Kit (Megazyme, Bray Business Park, Bray, Co., Wicklow, Ireland) according to the manufacturer’s instructions and following the standardized official AOAC procedure. Analyses were performed in triplicate, and results were expressed as grams of phytic acid per 100 g of sample.

#### 2.5.3. Total Antioxidant Capacity (TAC)

Prior to analysis, 0.1 g of each freeze-dried sample was extracted with 1 mL of a methanol/water solution (70:30, *v*/*v*), following the method described by Chiacchio et al. [30]. The mixture was vortexed and then centrifuged at 14,800 rpm for 10 min at 4 °C. The resulting supernatant was collected, filtered through 0.45 μm PTFE filters, and diluted fivefold before analysis.

TAC was determined on the soluble extracts using the colorimetric 2,2-diphenyl-1-picrylhydrazyl (DPPH) radical scavenging assay, as described by Hamzalioglu et al. [31]. The DPPH working solution was prepared by dissolving DPPH powder (Sigma-Aldrich, Milan, Italy) in methanol to obtain an absorbance of 0.9 ± 0.02 at 517 nm. The reaction was initiated by mixing 0.2 mL of the diluted extract with 1.0 mL of the DPPH working solution, followed by vortexing. After incubation for 10 min at room temperature in the dark the absorbance was measured at 517 nm using a UV/VIS spectrophotometer (PG Instruments, Wibtoft, UK). A blank was prepared by replacing the sample extract with the same volume of extraction solvent. Antioxidant activity was calculated as percentage DPPH radical inhibition according to the following equation:

% inhibition = [1 − (Abs of sample)/(Abs of blank)] * 100

TAC was quantified by comparison with a Trolox calibration curve (5–125 µM) and expressed as micromoles of Trolox equivalents (TE) per gram of dry matter. All extractions were performed in duplicate.

### 2.6. Chickpea-Based Fermented Beverage Production

Based on preliminary results, four selected LAB strains—*Pediococcus* (*P.*) *lolii* B72, *Lactiplantibacillus* (*Lactiplant.*) *plantarum* 95, *Lentilactobacillus* (*Lentilact.*) *diolivorans* 13-4A, and *Leuconostoc* (*Leuc.*) *mesenteroides* OM94—were used in combination with *Saccharomyces* (*S.*) *cerevisiae* LN7 to produce a fermented beverage from hydrolyzed chickpea flour. *Lactipl. plantarum* 95 and *S. cerevisiae* LN7 were also tested alone. Non-inoculated chickpea flour hydrolysates served as a control. All fermentation trials were performed in triplicate in a 100 mL volume. Inoculum rate (7 and 6 Log CFU/mL for bacteria and yeast, respectively) and fermentation conditions were the same as those reported in Section 2.5.1 Metabolite detection/quantification by HPLC and microbial counts (LAB and yeast) were carried out as described in Section 2.1 and Section 2.5.1, respectively. Viable yeast and LAB populations of beverage samples were assessed after cold storage (4–6 °C) for 30 days, also.

#### 2.6.1. Characterization of Chickpea-Based Beverages

TAC and total polyphenol content (TPC) were evaluated in chickpea-based beverages, both in the products themselves and in the digests obtained after in vitro digestion. Samples were freeze-dried before characterization. TAC was assessed as detailed in Section 2.5.3.

The determination of TPC was conducted on the same diluted extract used for the determination of TAC (Section 2.5.3), following the Folin–Ciocalteu method [32]. In a 15 mL Falcon tube, 0.5 mL of distilled water, 0.125 mL of the diluted extract, and 0.125 mL of Folin–Ciocalteu reagent (Sigma-Aldrich, Milan, Italy) were added. The resulting mixture was vortexed and left to react for 6 min. Then, 1.25 mL of 7.5% sodium carbonate (Sigma Aldrich, Milan, Italy) was added, and the reaction volume was brought to 3 mL with distilled water. After vortexing, the mixture was incubated in the dark at room temperature with gentle shaking for 90 min. The blank was prepared by replacing the sample extract with 0.125 mL of distilled water and following the same procedure. After incubation, absorbance was measured at 760 nm using a UV/VIS spectrophotometer (PG Instruments, Wibtoft, UK). Each sample was analyzed in triplicate, and the results were expressed as mg of gallic acid per 100 g of sample, based on a calibration curve prepared with a gallic acid standard.

#### 2.6.2. In Vitro Digestion

The gastrointestinal digestion of beverage samples was simulated using the INFOGEST in vitro digestion protocol [33]. Three sequential digestive phases were carried out: the simulated salivary phase (SSP), gastric phase (SGP), and intestinal phase (SIP). In the first digestion step (SSP), 2.5 g of each sample was combined with 2 mL of simulated salivary fluid (pH 7), 0.25 mL of amylase (1500 U/mL), 12.5 μL of CaCl_2_ (0.3 M), and 0.238 mL of water. Samples were incubated in a thermostatic water bath for 2 min at 37 °C with shaking at 160 rpm before proceeding to the gastric phase (SGP). After incubation, 4 mL of simulated gastric fluid (pH 3.0) and 2.5 μL of CaCl_2_ were added to each sample. The pH was adjusted to 3 using 0.250 mL of 1 M HCl, followed by the addition of 0.248 mL of water and 0.5 mL of pepsin (25,000 U/mL). Samples were incubated for 2 h at 37 °C with agitation at 130 rpm. For the SIP, 4.25 mL of simulated intestinal fluid (pH 7.0), 2.5 mL of pancreatin (800 U/mL), 1.25 mL of bile solution (160 mM), and 20 μL of CaCl_2_ were added. The pH was adjusted to 7.0 using 0.2 mL of 1 M NaOH and 1.78 mL of water, and samples were incubated for 2 h at 37 °C with shaking at 100 rpm. At the end of the intestinal phase, samples were centrifuged at 4000 rpm for 15 min at 4 °C to separate the supernatant (bioaccessible fraction) from the pellet (residual fraction). Both fractions were freeze-dried, and the polyphenol content (Section 2.6.1), along with the antioxidant activity (Section 2.5.3), was assessed on the bioaccessible fractions.

### 2.7. Statistical Analysis

Results were expressed as mean ± standard deviation. Significant differences between samples were explored by One-way ANOVA with *post hoc* Tukey test at the confidence level of *p* < 0.05 using the SPSS version 17 (SPSS Inc., Chicago, IL, USA). The relationship between TAC and TPC was evaluated by the Regression analysis using the MS-Excel data function.

## 3. Results and Discussion

### 3.1. LAB Microflora in Chickpea Soaking Water

Legume soaking is a multipurpose technological process that reduces the levels of antinutrients and improves the digestibility of beans. In addition, soaking promotes the breakdown of complex compounds, enhancing the bioavailability of beneficial nutrients [34]. Typically, the soaking process lasts between 12 and 36 h, depending on bean type. During soaking, the combination of elevated pH and nutrients released from the beans supports the proliferation of various microorganisms, including spore-forming bacteria (*Bacillus* and *Clostridium* spp.), *Enterobacteriaceae*, coliforms, staphylococci, LAB, yeast, and molds [34,35,36,37,38]. LAB often dominate the microbial community, reaching concentrations of 6–9 Log CFU/g and lowering the pH to 4.5–5.0 [35]. These bacteria produce lactic and acetic acids, which help inhibit the growth of spoilage and pathogenic microorganisms [39]. The use of LAB as starter cultures during soybean soaking for tempeh production has also been proposed [39].

**Figure 1:**
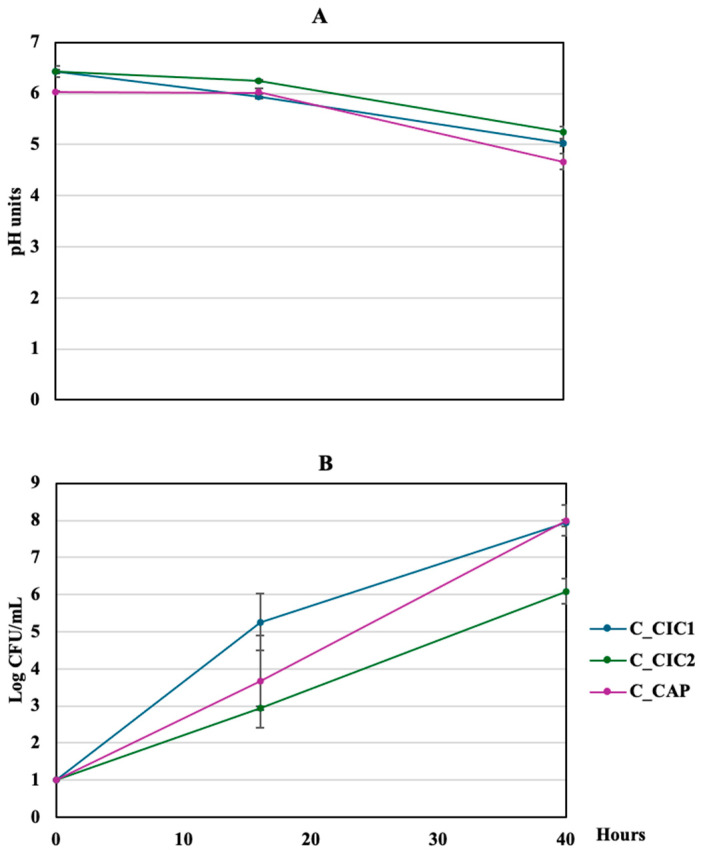
pH (**A**) and MRS counts (**B**) monitoring during chickpea seed soaking. CIC1 and CIC2, seeds from cultivar Cicerale. CAP, seeds from cultivar Caposele.

Two local chickpea cultivars were used in this study, yielding a total of three samples (CC1, CC2, and CAP) that were monitored during soaking. pH and MRS counts are reported in Figure 1.

During the first 16 h, which is the typical chickpea soaking time, pH slightly changes, even though MRS counts in sample CIC1 reached about 5 Log CFU/mL. However, after 40 h of incubation, counts significantly increased, reaching around 8 Log CFU/mL in samples CIC1 and CAP. Consequently, pH dropped below 5.5, with a minimum value in the CAP sample (4.67 ± 0.16). Spore-forming bacteria after 40 h of soaking were in the range of 2–3 Log CFU/mL.

This trend was further supported by HPLC monitoring of soluble sugars during soaking (Figure S1). At the beginning of the process (T0 h), both glucose and fructose were below the detection limit (<0.15 g/L each), but their concentrations increased to approximately 5 g/L after 16 h of soaking. Conversely, both sugars dropped to 2.5 g/L at the end of the monitoring period (T40 h). Regardless of the sample, the main metabolite detected was 2,3-butanediol with concentrations of 0.8 ± 0.05 and 1.0 ± 0.06 g/L at T16 h and T40 h, respectively. The presence of 2,3-butanediol is likely associated with the fermentative metabolism of *Bacillus* spp. [40].

Lactic acid was detected only after 40 h of incubation with concentrations of 0.56 ± 0.10 g/L in CIC1 and CIC2, and 0.98 ± 0.05 g/L in CAP. Similarly, acetic acid was detected only at T40 h, ranging from 0.13 ± 0.03 g/L to 0.32 ± 0.05 g/L, while succinic acid was found exclusively in CIC samples (0.45 ± 0.06 g/L).

No presumptive LAB colonies were recovered from the counting plates of samples CIC2 and CAP at T16 h, as most colonies were identified microscopically as spore-forming bacteria. Six isolates (two per sample) were obtained and identified: five strains were assigned to *Bacillus* (*B.*) *velezensis*/*amyloliquefaciens*, and one strain (from CIC2) to *Peribacillus simplex*, as determined by MALDI-TOF analysis.

At T40 h, the counting plates were predominantly populated by presumptive LAB. Seven isolates were identified by MALDI-TOF as *Weissella* (*W.*) *cibaria*/*confusa* (three isolates, one from each sample), *Lactiplant. plantarum* (one from CIC1 and one from CIC2), W. *paramesenteroides* (from CIC2), and *P. pentosaceus* (from CAP). Partial 16S rDNA sequencing confirmed the species identification.

Furthermore, in this study, a clear microbial succession from *Bacillus* spp. to LAB was observed. *Bacillus velezensis*/*amyloliquefaciens*, known for its amylolytic activity, likely contributed to the release of oligosaccharides and fermentable sugars from starch, thereby supporting lactic acid fermentation by LAB in the second phase.

Previous studies [34,35,36,37,38,39,40,41,42] have reported the presence of different LAB species from genera such as *Lactobacillus*, *Pediococcus*, *Leuconostoc*, *Lactococcus*, *Streptococcus*, and *Enterococcus* spp. during legume soaking. However, the high occurrence (up to 8 Log CFU/mL) of *Weissella* spp. observed in this study has not been previously documented in this ecosystem. High-throughput sequencing analysis has detected *Weissella* spp. on soybeans after 18 h of soaking but not in the soaking water [37]. Moreover, *Weissella* spp. has been identified as the dominant LAB in the chickpea-based liquid starters used for bread production [43]. As recently reviewed [44], *Weissella* spp. strains play important roles in food preservation and functionality, making them promising candidates for innovative starter cultures [44,45].

In this work, two *W. cibaria*/*confusa* strains (76 and 113) showed noteworthy technological traits, including high EPS production and antimicrobial activity against *Staph. aureus* DSM799 (Table S1). Regarding their physiological features, all strains were able to assimilate fructose and maltose, while none showed urease or amylolytic activity, or produced the biogenic amines tyramine or histamine. None of the autochthonous strains could utilize raffinose, despite this ability being common among strains isolated from various food ecosystems. For instance, *Lentilact. diolivorans* strains (13-1B and 13-4A), *Lacticas. casei* LBC491, and *Leuc*. *mesenteroides* exhibited raffinose utilization (Table S1). Conversely, maltodextrin utilization was uncommon: aside from moderate growth in *Lacticas. casei* LBC491, only the strain *Lactiplant. plantarum* 95 (isolated from chickpea soaking water) displayed this capability (Table S1). Overall, chickpea soaking water appears to be a valuable reservoir of biotechnologically relevant microorganisms with potential applications beyond traditional food fermentations.

### 3.2. Characterization of Fermented Chickpea Flour Hydrolysates

Plant-based beverages, including plant-based milk alternatives, are defined as water extracts obtained from vegetable raw materials [15,16,17,18,19,20,21,22,23,24,25,26,27,28,29,30,31,32,33,34,35,36,37,38,39,40,41,42,43,44,45,46]. In the case of legumes, the primary raw material consists of seeds. The main steps in pulse-based beverage production typically include seed soaking in water (1:3–1:5 *w*/*v*), cooking in water, milling and homogenization in water, sieving or filtering, and thermal treatment (pasteurization or sterilization) [14,15,16,17,18,19,20,21,22,23,24,25,26,27,28,29,30,31,32,33,34,35,36,37,38,39,40,41,42,43,44,45,46,47,48,49,50,51,52]. An additional germination step following soaking has also been proposed by Lopes et al. [53].

However, this conventional process generates both liquid (soaking and cooking waters) and solid (sieving/filtering residues) waste and often yields beverages unsuitable for fermentation due to their low content of fermentable sugars. To address this, sugars are commonly added before thermal treatment in legume-based beverage formulations [14,15,16,17,18,19,20,21,22,23,24,25,26,27,28,29,30,31,32,33,34,35,36,37,38,39,40,41,42,43,44,45,46,47,48,49,50,51,52]. Ritter et al. [54] proposed the malting and mashing of lupin and faba bean using commercial enzymes to produce fermentable substrates. More recently, plant-based beverages have been developed from rice flour [55], einkorn (*Triticum monococcum* L. ssp. *monococcum*) [56], chickpea/carob pulp blend [57], and quinoa/chickpea mixtures [48]. In these studies, Maoloni et al. [56] supplemented sugars to initiate fermentation, whereas Khan et al. [55] applied enzymatic digestion to release fermentable carbohydrates.

In the present study, chickpea flour and enzymatic hydrolysis were used to prepare the basal medium for fermentation. Hydrolysis efficiency was confirmed by HPLC monitoring of reducing sugars. As expected from the flour composition (3.5% *w*/*w* soluble sugars), glucose and fructose at the beginning of the hydrolysis were 3.2 ± 0.15 g/L (Figure S2A), increasing to 12.75 ± 0.49 g/L by the end of the process (Figure S2B). Due to glucoamylase activity, glucose represented the predominant monosaccharide (Figure S2B).

Legume-based beverages can be produced with or without fermentation [15]. Typical fermentation starters include LAB, *Bacillus* spp., and yeasts (e.g., *Saccharomyces* spp.) [15]. However, as recently reported by Chiacchio et al. [10], many LAB-mediated effects on the nutritional and functional properties of fermented legume products are strain-specific. Accordingly, 11 different strains, comprising 9 LAB (4 isolated in this study) and 2 yeasts (MP24 and LN7), were used to ferment the chickpea hydrolysates.

**Figure 2:**
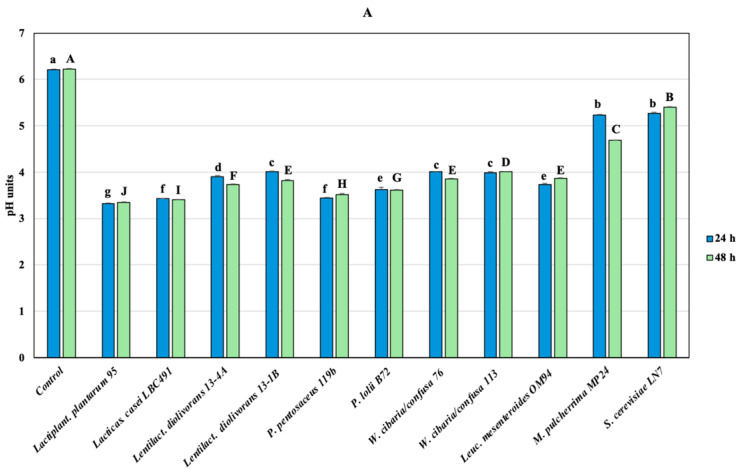

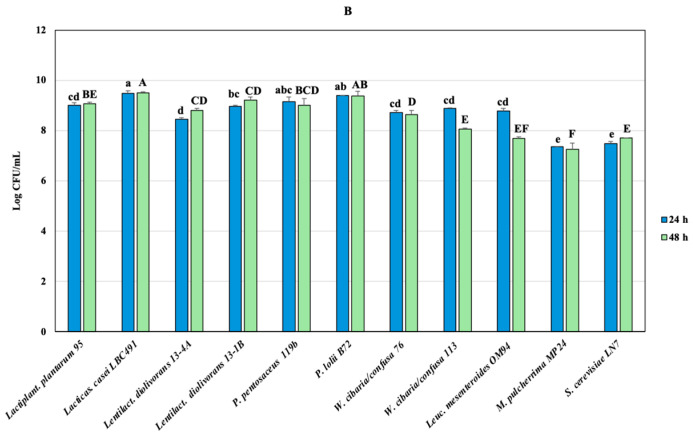
pH (**A**) and microbial counts (**B**) changes in hydrolyzed chickpea flour fermented by different microorganisms after 24 and 48 h. Data are reported as mean ± sd. Significant differences were assessed by one-way ANOVA and Tukey’s *post hoc* test (*p* < 0.05). Different letters represent significant differences: uppercase, at 24 h; lowercase, at 48 h.

Despite not being sterile, the pH of the uninoculated hydrolysates (Control) remained stable at approximately 6.1 throughout the incubation period. All LAB strains lowered the pH below 4 within 24 h, while *M. purcherrima* (MP24) caused a gradual pH reduction, which reached 4.69 in 48 h (Figure 2A).

The sharp pH drop may explain the two-log decline observed in the populations of strains 113 (*W. cibaria*) and OM94 (*Leuc. mesenteroides*) after 24 h (Figure 2B). Conversely, both *Lentilac. diolivorans* strains (13-1B and 134A) exhibited a one-log population increase during this interval, consistent with the typical slow growth rate of this genus [58].

Regarding sugar utilization, *S. cerevisiae* and *M. pulcherrima* were the most efficient fermenting strains (Table 2). The observed pH decrease in yeast-fermented hydrolysates was likely associated with succinic acid production. In contrast, bacterial strains consumed less sugar overall but caused a greater pH reduction due to lactic acid formation. *Lacticas. casei* LBC491 and *Lactiplant. plantarum* 95 efficiently fermented sugars, producing lactic acid as the sole organic acid. *W. cibaria* (strains 76 and 113) and *Lentilact. diolivorans* (13-4A and 13-4B) produced lactic acid along with acetic acid and ethanol, consistent with their heterofermentative metabolism. Conversely, the heterofermentative *Leuc. mesenteroides* (OM94) did not generate acetic acid but accumulated notable amounts of succinic acid (Table 2). The formation of succinic acid from citrate or malate through the reductive TCA pathway has previously been described for several LAB genera, including *Leuconostoc* spp. [59,60].

**Figure 3:**
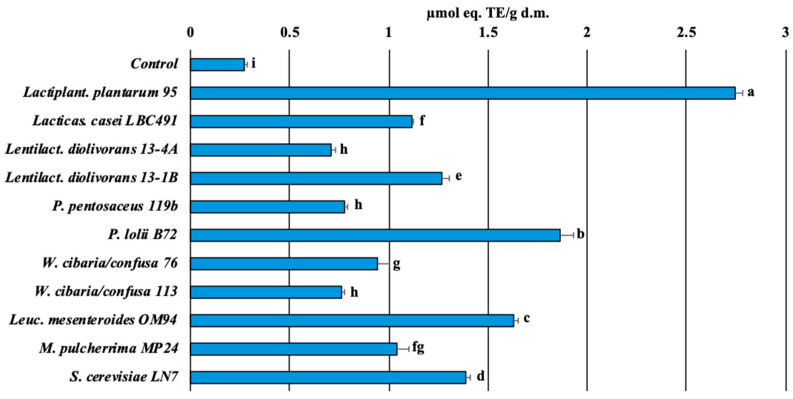
Total Antioxidant Capacity (TAC) of hydrolyzed chickpea flour fermented with different microorganisms, expressed as µmol Trolox equivalents (TE)/g dry matter (d.m.). Different letters on the bars indicate significant differences among samples (*p* < 0.05), as determined by one-way ANOVA followed by Tukey’s *post hoc* test.

**Figure 4:**
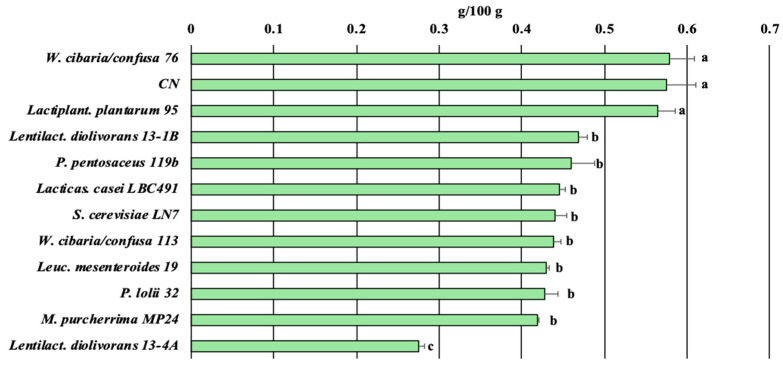
Phytic acid content (g/100 g) in chickpea flour hydrolyzed after a 48 h fermentation. Different letters on the bars represent significant differences among the samples (*p* < 0.05), as determined by one-way ANOVA followed by Tukey’s *post hoc* test.

The eleven fermented chickpea hydrolysates and the controls were evaluated for TAC and phytic acid content. *Lactiplant. plantarum* 95 promoted the highest TAC with a value exceeding 2.5 µmol Trolox/g, followed by *P. lolii* B72, *S. cerevisiae* LN7, and *Leuc. mesenteroides* OM94 (Figure 3). By contrast, *Lactiplant. plantarum* 95 and *W. cibaria* 76 could not reduce the phytic acid content of the fermented product compared to the control (Figure 4). The unique LAB strain that significantly reduced the phytic acid content (about 50% less than the control) was *Lentilact. diolivorans* 13-4A.

### 3.3. Chickpea-Based Fermented Beverage Evaluation

TAC (Figure 3), acidification (Figure 2A), growth performance (Figure 2B), residual sugar content (Table 2), phytic acid degradation (Figure 4), and overall metabolic activities (Table S1) were used as criteria for selecting the strains employed in chickpea-based fermented beverage production (Table 3). Specifically, strains 95, OM94, and B72 were characterized by high TAC activity, while strain 13-4A expressed fair phytasic activity. Additionally, strains 95 and B72 showed antagonistic activity against *E. hirae*, while strain OM94 was an EPS-producer and a raffinose-fermenting bacterium, an interesting feature shared by 13-4A too.

As reviewed by Mefleh et al. [15], mixed starter cultures are generally more effective than single strains. For instance, LAB/yeast combinations have proven particularly suitable for fermenting peanut-soy-based matrices [61] and extruded brown rice substrates [55]. However, the literature regarding the use of yeasts in chickpea fermentation remains limited [62]. Recently, *Debaryomyces hansenii*, *Yarrowia lipolytica* and *S. cerevisiae* were used to ferment hydrated chickpea flour, and the results showed that these yeasts can enhance the nutritional and functional properties of chickpea flour [62]. Nevertheless, since yeasts produce low amounts of acid during fermentation, their use under unsterile conditions does not adequately inhibit background microflora and spoilage bacteria (*Enterobacteriaceae*) [62]. In this context, the selected LAB strains were co-cultured with *S. cerevisiae* LN7. Across all trials, the pH reached its minimum within the first 24 h of fermentation and remained nearly constant thereafter (Table 3).

Samples inoculated only with yeast (LN7) held the highest pH (4.95 ± 0.05), whereas those inoculated solely with *Lactiplant. plantarum* (95) reached the lowest (3.19 ± 0.01). Significant differences were observed among the single LAB/yeast combinations (Table 3), with the lowest pH values (3.30–3.40) in those containing homofermentative LAB (*P. lolii* B72 and *Lactiplant. plantarum* 95). These results differ from those reported by Ustaoğlu-Gençgönül et al. [52], where chickpea beverages produced with kefir grains showed a maximum pH drop from 6.46 to 6.89 to 4.52–4.70.

LAB and yeast counts monitored at 24 h intervals are reported in Table 3. Initial LAB counts were all approximately 7 Log CFU/mL for all samples and increased by 1–3 Log CFU units after 24 h of fermentation (T24), depending on the microbial combination. By the end of the monitoring period (T48 h), four samples (LN7/B72, LN7/13-4A, LN7/95, and 95) reached LAB counts of approximately 9 Log CFU/mL. In contrast, the LN7/OM94 combination (*S. cerevisiae*/*Leuc. mesenteroides*) showed a different trend with a moderate increase (~1.2 Log CFU units) during the first 24 h, followed by a decrease to levels comparable to the initial inoculum at 48 h (Table 3). Overall, LAB concentrations observed in this study were higher than those previously reported for chickpea beverages by Duarte et al. [47] and Ustaoğlu-Gençgönül et al. [52].

Yeast growth showed more variable trends among samples (Table 3). The strain LN7 reached counts up to 7 Log CFU/mL both when inoculated alone and in combination with OM94. In other combinations, yeast growth was negligible (LN7/95 and LN7/B72) or not detected (LN7/13-4A). In the LN7/13-4A combination, yeast counts decreased by approximately 3 log CFU/mL over 48 h, suggesting competitive interactions between *Saccharomyces cerevisiae* LN7 and *Lentilact. diolivorans*. Similarly, *Lentilact. diolivorans* 13-4A, *Lactiplant. plantarum* 95, and *P. lolii* B72 appeared to limit yeast growth, while LN7 negatively affected the persistence of *Leuc. mesenteroides* OM94.

At the end of fermentation (T48), 3 beverage samples (LN7/B72, LN7/13-4A, LN7/95) exhibited high LAB counts (>9 Log CFU/mL) together with detectable yeast populations (≥3 log CFU/mL) (Table 3). In addition, LAB populations in LN7/13-4A and LN7/95 remained stable after 30 days of refrigerated storage (4–6 °C) (Figure S3A). The LN7/OM94 retained approximately 7 Log CFU/mL of both LAB and yeasts after the same storage period (Figure S3). These results demonstrate the ability of selected microbial combinations to maintain viability during fermentation and cold storage. While the presence of viable microorganisms is a prerequisite for potential functional applications, further in vivo studies are required to assess their effects on gut microbiota composition or host health. These findings suggest that including chickpea beverages in the diet may support health by providing a consistent intake of harmless live microorganisms [63]. Diets rich in fermented foods have been associated with increased gut microbiota diversity and reduced inflammatory markers [64].

Beverage samples also differed in their chemical composition, including residual sugars, acids, glycerol, and ethanol content (Table 4).

All fermented samples, except LN7/13-4A, retained less than 1 g/L of free sugars. Yeast (LN7) led to the production of ethanol, succinic acid, and glycerol, while the beverage produced by using *Lactiplant. plantarum* 95 alone or in combination with yeast (LN7/95), as well as the combination LN7/B72, mainly contained lactic acid (Table 4). The sample LN7/13-4A was characterized by a lactic acid/ethanol mix. Succinic acid was retrieved in the sample LN7/OM94, confirming the results obtained by using this LAB strain alone (Table 2).

### 3.4. Total Antioxidant Capacity and Total Polyphenol Content of the Chickpea-Based Beverages

**Figure 5:**
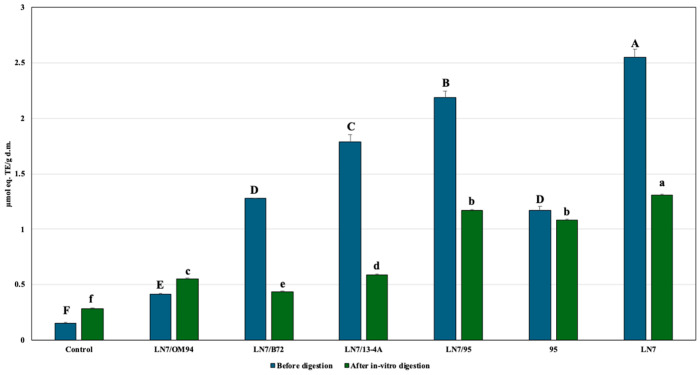
Total Antioxidant Capacity (TAC) of chickpea-based fermented beverages before digestion and of the corresponding bioaccessible fractions after in vitro digestion, compared with the Control (unfermented chickpea-based beverage). LN7/OM94: *S. cerevisiae*/*Leuc. mesenteroides*; LN7/B72: *S. cerevisiae*/*P. lolii*; LN7/13-4A: *S. cerevisiae*/*Lentilact. diolivorans*; LN7/95: *S. cerevisiae*/*Lactiplant. plantarum*; 95: *Lactiplant. plantarum*. Different letters represent significant differences (*p* < 0.05): uppercase letters refer to samples before digestion, while lowercase letters refer to samples after in vitro digestion.

Figure 5 shows the TAC of the beverages and the bioaccessible fractions collected at the end of the in vitro digestion. The beverage obtained through fermentation with *S. cerevisiae* alone (LN7) exhibited the highest TAC both in the beverage and after digestion, followed by the LN7/95 combination. These results are consistent with those reported by Handa et al. [65] who observed undetectable TAC in the control and increases ranging from 0.1 to-0.15 μmol CatEq/mL in chickpea-based milk alternatives fermented with *Lactiplant. pentosus*, *Lactococcus lactis*, or a combination of the two strains.

Although TAC remained higher than in the control for nearly all samples, it generally decreased after digestion. The smallest loss was observed in beverages fermented solely with *Lactiplant. plantarum* 95. Conversely, the LN7/OM94 combination (*S. cerevisiae*/*Leuc. mesenteroides*) exhibited a significant increase in TAC following digestion (Figure 5).

**Figure 6:**
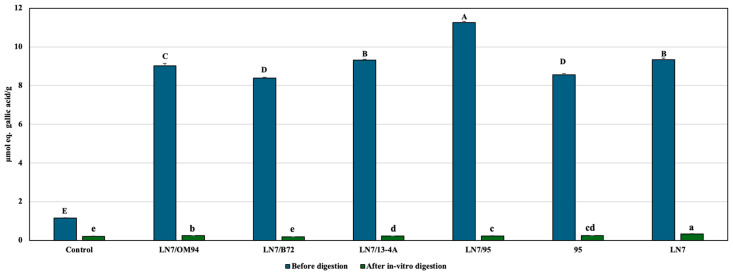
Total Polyphenol Content (TPC) of chickpea-based beverages before digestion and of the corresponding bioaccessible fractions after in vitro digestion, compared with the control (unfermented chickpea-based beverage). LN7/OM94: *S. cerevisiae*/*Leuc. mesenteroides*; LN7/B72: *S. cerevisiae*/*P. lolii*; LN7/13-4A: *S. cerevisiae*/*Lentilact. diolivorans*; LN7/95: *S. cerevisiae*/*Lactiplant. plantarum*; 95: *Lactiplant. plantarum*. Different letters represent significant differences (*p* < 0.05): uppercase letters refer to samples before digestion, while lowercase letters refer to samples after in vitro digestion.

A reduction in TAC after in vitro digestion has also been reported in apple cider [66]. Cavia et al. [66] suggested that changes in acid-base ratios during digestion may affect DPPH radical reactivity, which could explain the TAC decrease observed in beverages LN7, LN/32, LN7/93, and LN7/95. Conversely, the slight post-digestion increases in TAC detected in beverage LN7/OM94 (*S. cerevisiae*/*Leuc. mesenteroides*) and in the control may result from the release of antioxidant compounds following digestion-induced matrix breakdown. The TPC of all beverage samples before digestion was significantly higher than that of the control (Figure 6). The highest TPC (11.25 ± 0.059 µmoL eq gallic acid/g) was recorded in LN7/95 (*S. cerevisiae*/*Lactiplant. plantarum*), followed by LN7/13-4A (*S. cerevisiae*/*Lentilact. diolivorans*) and *Lactiplant. plantarum* 95 alone (Figure 6).

In plant-based foods, fermentation can enhance the release of antioxidants and phenolic compounds from the matrix by disrupting the plant cell walls [67].

After digestion, TPC decreased markedly in all samples (Figure 6). The LN7/B72 combination showed a value (0.18 ± 0.01 µmoL eq gallic acid/g) close to that of the control (0.21 ± 0.00 µmoL eq gallic acid/g). In all other samples, the TPC post-digestion values exceeded the control. The trial LN7 (yeast only) reached the highest value (0.33 ± 0.01 µmoL eq gallic acid/g). Polyphenols may interact with other components, be metabolized, or degraded during digestion, making their fate critical for assessing bioaccessibility [68].

A pronounced decrease in TPC after digestion has also been reported in herbal infusions [68] and apple ciders [66], where bioaccessibility and bioactivity were influenced by pH changes and enzymatic activities during digestion.

The relationship between TAC and TPC in the beverages and in the corresponding bioaccessible fractions obtained after in vitro digestion was explored by regression analysis (Figure S4A,B). In the beverage samples (Figure S4A), the regression yielded a coefficient of determination (R^2^ = 0.4922), indicating that approximately 49.2% of the variability in TAC could be statistically associated with TPC. In the bioaccessible fractions after digestion (Figure S4B), 55.6% of the variability in TAC was associated with TPC. These results suggest that polyphenols contribute to antioxidant capacity in both the beverages and their bioaccessible fractions. However, TPC alone does not fully account for the observed TAC. Other bioactive compounds, such as peptides, organic acids, and fermentation-derived products, are also likely to contribute to the overall antioxidant capacity. These findings underscore the multifactorial nature of antioxidant compound content and bioaccessibility in chickpea-based beverages.

## 4. Conclusions

The increasing demand for non-dairy alternatives provides an opportunity to develop food products that are both health-promoting and environmentally sustainable. In this study, a novel, sustainable process was developed for the production of a functional chickpea-based beverage.

The process is based on the enzymatic hydrolysis of chickpea flour to generate fermentable sugars in situ, eliminating the need for added sugars and improving process sustainability. The use of chickpea flour instead of whole seeds simplifies production, reduces waste, and lowers water and energy consumption by avoiding multiple filtration steps. Moreover, the use of a standardized flour enhances process reproducibility and ensures greater control of fermentation parameters, which is particularly relevant for industrial scale-up.

An optimal fermentation time of 24 h was identified, beyond which no further technological and functional improvements were observed. Among the tested microbial cultures, the co-culture of *Saccharomyces cerevisiae* LN7 and *Lactiplant. plantarum* 95 showed the best performance, promoting microbial growth and significantly increasing TAC and TPC of the beverage compared to non-fermented controls. Although TAC and TPC values decreased after in vitro digestion, they remained higher than those of the controls, indicating good stability and bioaccessibility of bioactive compounds. The present study primarily focused on technological, nutritional, and functional aspects of the beverage. Sensory attributes, which are crucial for consumer acceptance and product success, were not evaluated and represent a limitation of this work. Nevertheless, the standardized and scalable process developed here, together with the identified microbial combinations, provides a solid basis for future studies aimed at sensory optimization and formulation refinement.

Overall, this study demonstrates the feasibility of producing a sustainable, chickpea-based fermented beverage with functional properties and viable microorganisms. The results underscore the importance of strain selection and process optimization in the development of next-generation plant-based foods. Finally, some interesting technological features (raffinose fermentation, EPS production, antimicrobial activity) harbored by LAB strains used in the first phase of this study may be profitably exploited for designing specific plant-based fermented foods in the future.

## Figures and Tables

**Table 1:** Microorganisms used during this study and the source of isolation.

Taxon	Strain Code	Source [Reference]
*Lactiplantibacillus plantarum*	95	Chickpea (cv. Cicerale) soaking water (This study)
*Lacticaseibacillus casei*	LBC491	Provolone del Monaco Cheese [19]
*Lentilactobacillus diolivorans*	13-4A	Fermented sheep milk (Gioddu) (Unpublished)
*Lentilactobacillus diolivorans*	13-1B	Fermented sheep milk (Gioddu) (Unpublished)
*Pediococcus pentosaceus*	119b	Chickpea (cv. Caposele) soaking water (This study)
*Pediococcus lolii*	B72	Sourdough [20]
*Weissella cibaria/confusa*	76	Chickpea (cv. Cicerale) soaking water (This study)
*Weissella cibaria/confusa*	113	Chickpea (cv. Caposele) soaking water (This study)
*Leuconostoc mesenteroides*	OM94	Fermented table olives [21]
*Metschnikowia pulcherrima*	MP24	Falanghina grapes (Unpublished)
*Saccharomyces cerevisiae*	LN7	Sourdough (Unpublished)

**Table 2:** Final pH (T48 h), residual sugars (glucose + fructose), succinic acid, lactic acid, acetic acid, glycerol, and ethanol of fermented (48 h) hydrolysed chickpea flour by different LAB and yeasts. Data are shown as mean ± sd. HPLC data are expressed in g/L. Different lowercase letters indicate differences between samples at the same incubation time. Significant differences were assessed by One-way ANOVA and Tukey’s *post hoc* test (*p* < 0.05).

Strain	pH 48 h	Residual Sugars	Succinic Acid	Lactic Acid	Acetic Acid	Glycerol	Ethanol
Control	6.22 ± 0.01 _a_	12.75 ± 0.49 _a_	nd *	nd	nd	nd	nd
*Lactiplant. plantarum* 95	3.35 ± 0.01 _j_	2.15 ± 0.49 _d_	nd	13.10 ± 0.04 _a_	nd	nd	nd
*Lacticas. casei* LBC491	3.41 ± 0.00 _i_	1.39 ± 0.03 _d,e_	nd	11.47 ± 0.04 _b_	nd	nd	nd
*Lentilact. diolivorans* 13-4A	3.73 ± 0.01 _f_	3.69 ± 0.23 _c_	nd	5.55 ± 0.09 _e,f_	0.66 ± 0.21 _b_	nd	2.83 ± 0.04 _c_
*Lentilact. diolivorans* 13-4B	3.82 ± 0.02 _e_	3.51 ± 0.41 _c_	nd	4.99 ± 0.05 _f_	1.88 ± 0.04 _a_	nd	2.40 ± 0.03 _d_
*P. pentosaceus* 119b	3.51 ± 0.03 _h_	5.72 ± 0.16 _b_	nd	8.41 ± 0.29 _d_	nd	nd	nd
*P. acidilactici* B72	3.61 ± 0.01 _g_	5.00 ± 0.14 _b_	nd	9.95 ± 0.13 _c_	nd	nd	nd
*W. cibaria/confusa* 76	3.86 ± 0.01 _e_	4.55 ± 0.36 _b,c_	nd	5.66 ± 0.17 _e_	0.66 ± 0.06 _b_	nd	1.30 ± 0.07 _g_
*W. cibaria/confusa* 113	4.01 ± 0.01 _d_	5.45 ± 0.21 _b_	nd	4.13 ± 0.11 _g_	0.31 ± 0.09 _c_	nd	1.96 ± 0.06 _e_
*Leuc. mesenteroides* OM94	3.87 ± 0.01 _e_	1.42 ± 0.05 _d,e_	1.02 ± 0.05 _a_	3.14 ± 0.09 _h_	nd	nd	1.51 ± 0.06 _f_
*M. pulcherrima* MP24	4.69 ± 0.01 _c_	0.38 ± 0.03 _e_	0.43 ± 0.10 _b_	nd	nd	1.34 ± 0.09 _a_	3.71 ± 0.15 _b_
*S. cerevisiae* LN7	5.40 ± 0.01 _b_	0.92 ± 0.08 _e_	0.40 ± 0.20 _b_	nd	nd	1.52 ± 0.65 _a_	6.03 ± 0.13 _a_

* nd: not detected.

**Table 3:** pH, LAB, and yeast counts (Log CFU/mL) in chickpea-based beverages at 0, 24, and 48 h of fermentation. Data are reported as mean ± sd. Different lowercase letters indicate significant differences among samples at the same incubation time (*p* < 0.05).

Beverages *	pH			LAB Counts			Yeast Counts		
	0 h	24 h	48 h	0 h	24 h	48 h	0 h	24 h	48 h
LN7/OM94	6.21 ± 0.00 _a_	4.07 ± 0.01 _b_	4.05 ± 0.01 _b_	6.72 ± 0.12 _a_	7.96 ± 0.22 _b_	6.88 ± 0.09 _b_	5.26 ± 0.05 _a_	6.44 ± 0.04 _b_	6.72 ± 0.12 _a_
LN7/B72	6.10 ± 0.01 _b_	3.54 ± 0.01 _c_	3.40 ± 0.01 _d_	6.75 ± 0.05 _a_	9.51 ± 0.02 _a_	9.56 ± 0.07 _a_	5.51 ± 0.45 _a_	5.59 ± 0.11 _c_	4.93 ± 0.17 _b_
LN7/13-4A	6.08 ± 0.04 _b_	4.05 ± 0.07 _b_	3.70 ± 0.08 _c_	6.83 ± 0.16 _a_	9.41 ± 0.04 _a_	9.26 ± 0.24 _a_	5.83 ± 0.16 _a_	5.69 ± 0.21 _c_	3.36 ± 0.32 _c_
LN7/95	6.05 ± 0.01 _b_	3.55 ± 0.07 _c_	3.30 ± 0.02 _d_	6.67 ± 0.36 _a_	8.10 ± 0.17 _b_	9.32 ± 0.28 _a_	5.68 ± 0.16 _a_	5.00 ± 0.00 _d_	4.46 ± 0.15 _b_
95	6.09 ± 0.01 _b_	3.49 ± 0.02 _c_	3.19 ± 0.01 _d_	6.83 ± 0.33 _a_	8.22 ± 0.27 _b_	9.52 ± 0.24 _a_	nd	nd	nd
LN7	6.22 ± 0.01 _a_	5.46 ± 0.08 _a_	4.95 ± 0.05 _a_	nd **	nd	nd	5.40 ± 0.20 _a_	7.16 ± 0.28 _a_	7.33 ± 0.35 _a_

* LN7/OM94: *S. cerevisiae*/*Leuc. mesenteroides*; LN7/B72: *S. cerevisiae*/*P. lolii*: LN7/13-4A: *S. cerevisiae*/*Lentilact. diolivorans*; LN7/95: *S. cerevisiae*/*Lactiplant. plantarum*; LN7: *S. cerevisiae*; 95: *Lactiplant. plantarum*. ** nd: not detected.

**Table 4:** pH and residual sugars (glucose + fructose) (Rs), succinic acid (Sa), lactic acid (La), glycerol (Gly), and ethanol (Eth) of fermented (48 h) chickpea-based beverage. All data are expressed in g/L. Data are shown as mean ± sd. Different lowercase letters indicate significant differences among the samples assessed by one-way ANOVA and Tukey’s *post hoc* test (*p* < 0.05).

Samples *	pH	Rs	Sa	La	Gly	Eth
**CN**	6.22 ± 0.01 _a_	12.14 ± 0.23 _a_	nd **	nd	nd	nd
**LN7/OM94**	4.08 ± 0.04 _c_	0.70 ± 0.14 _c_	1.49 ± 0.14 _a_	4.85 ± 0.35 _b_	nd	4.05 ± 0.35 _b_
**LN7/B72**	3.43 ± 0.04 _e_	0.54 ± 0.08 _c,d_	0.18 ± 0.01 _c_	11.01 ± 0.17 _a_	nd	nd
**LN7/13-4A**	3.72 ± 0.03 _d_	2.07 ± 0.33 _b_	nd	4.00 ± 0.28 _b_	nd	3.20 ± 0.08 _c_
**LN7/95**	3.29 ± 0.01 _e,f_	0.30 ± 0.02 _c,d_	nd	11.40 ± 0.14 _a_	nd	nd
**95**	3.22 ± 0.02 _f_	0.60 ± 0.08 _c,d_	0.16 ± 0.05 _c_	11.7 ± 0.28 _a_	nd	nd
**LN7**	4.92 ± 0.04 _b_	0.08 ± 0.04 _d_	0.39 ± 0.03 _b_	nd	0.39 ± 0.2	4.94 ± 0.08 _a_

* CN: Negative control at time 0; LN7/OM94: *S. cerevisiae*/*Leuc. mesenteroides*; LN7/B72: *S. cerevisiae*/*P. lolii*: LN7/13-4A: *S. cerevisiae*/*Lentilact. diolivorans*; LN7/95: *S. cerevisiae*/*Lactiplant. plantarum*; LN7: *S. cerevisiae*; 95: *Lactiplant. plantarum*. ** nd: not detected.

## Data Availability

The original contributions presented in this study are included in the article/Supplementary Materials. Further inquiries can be directed to the corresponding author.

## Supplementary Materials

The following supporting information can be downloaded at: https://www.mdpi.com/article/10.3390/foods15030523/s1. Table S1, Features of technological interest for LAB strains used in the present study; Figure S1, HPLC chromatograms of soaking water: (A) T0 h; (B) T16 h; (C) T40 h; Figure S2, Change in monosaccharide (glucose and fructose) concentration during chickpea flour hydrolysis: (A) beginning; (B) end (after thermal treatment at 100 °C); Figure S3, LAB (panel A) and yeast (panel B) counts (Log CFU/mL) of chickpea-based beverages samples after cold storage (4–6 °C) for 30 days; Figure S4, Regression analysis between total antioxidant capacity (TAC) and total polyphenol content (TPC) of chickpea-based fermented beverages (panel A) and the bioaccessible fractions collected after in vitro digestion (panel B).
