# Supplementary material for: Chemical and Functional Properties of Chickpea (Cicer arietinum L.)-Based Fermented Beverages Produced Using Different Lactic Acid Bacteria
Source: Foods. 2026 Feb 3;15(3):523. doi: 10.3390/foods15030523 (PMC12896596; doi:10.3390/foods15030523)
Supplement: Supplementary file 1 [file foods-15-00523-s001.zip › foods-4048048-supplementary.pdf]

Chemical and functional properties of kefir-like chickpea (Cicer arietinum L.) beverages produced by using different lactic acid bacteria

Angela Pazzanese[1], Silvia Tagliamonte[1], Maria Aponte[1], Giuseppe Blaiotta[1]*, Manuela Flavia Chiacchio[1], Prakriti Khanal[1], Paola Vitaglione[1]

Supplementary Materials

Table S1. Features of technological interest for LAB strains used in the present study.

Strain	Fermentation					EPS production		Antagonistic activity				
	Maltose	Raffinose	Arabinose	Saccharose	Maltodextri	mMRS	MDIA	*P. roqueforti*	*B. coagulans*	*S. aureus*	*E. hirae*	*E. coli*
Lactiplant. plantarum 95	+	-	-	+	+	-	-	+	22[a]	±[b]	+	-
Lacticas. casei LBC491	+	±	±	±	±	±	±	+	22	-	+	-
Lentilact. diolivorans 13-4A	+	+	-	-	-	-	-	+	14	-	-	-
Lentilact. diolivorans 13-1B	+	+	-	-	-	-	-	+	16	-	-	-
P. pentosaceus 119b	+	-	-	-	-	-	-	+	2	-	+	-
P. lolii B72	-	-	-	-	-	-	-	+	18	-	+	-
W. cibaria/confusa 76	+	-	-	+	-	++	++	+	18	+	-	-
W. cibaria/confusa 113	+	-	-	+	-	++	++	+	28	+	+	-
Leuc. mesenteroides OM94	+	+	-	+	-	+	+	+	20	-	-	-

[a] Diameter (mm) of inhibition halos surrounding spots after a further 24 h of incubation at 37°C.

[b] +: Inhibition halos larger than 1 mm; ±: Inhibition halos < 1 mm; -: no Inhibition halo.

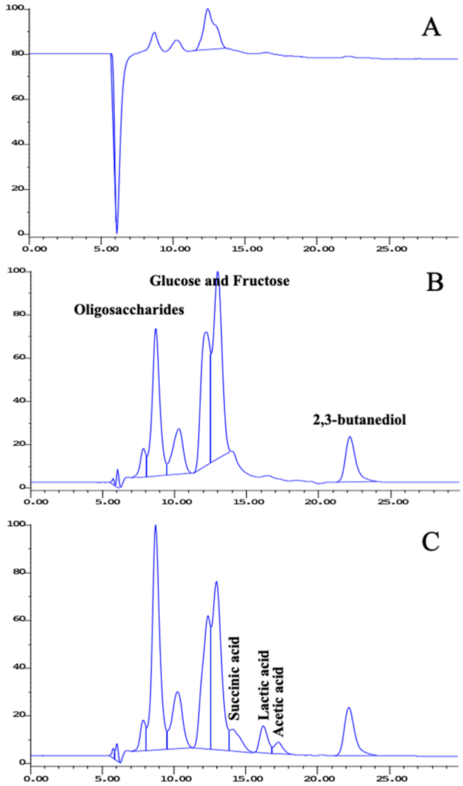

Figure S1. HPLC chromatograms of soaking water: A) T0h; B) T16h; C) T40 h.

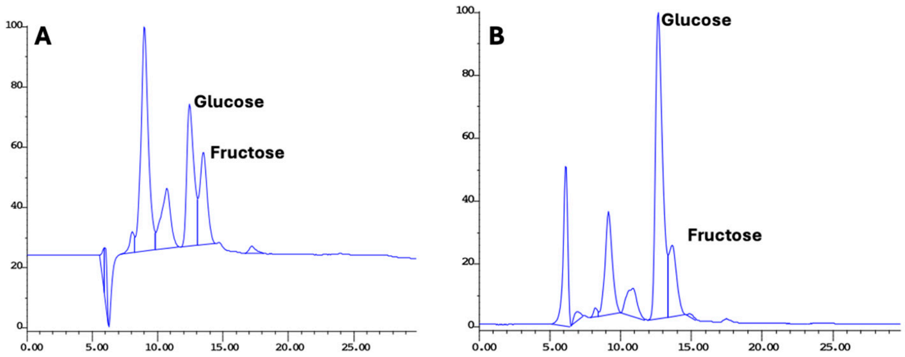

Figure S2. Change of monosaccharide (glucose and fructose) concentration during chickpea flour hydrolysis: A) beginning; B) end (after thermal treatment at 100°C).

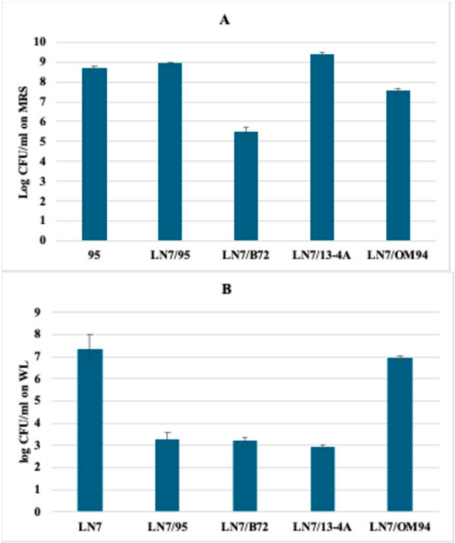

Figure S3. LAB (panel A) and yeast (panel B) counts (Log CFU/mL) of chickpea-based beverages samples after cold storage (4-6 °C) for 30 days.

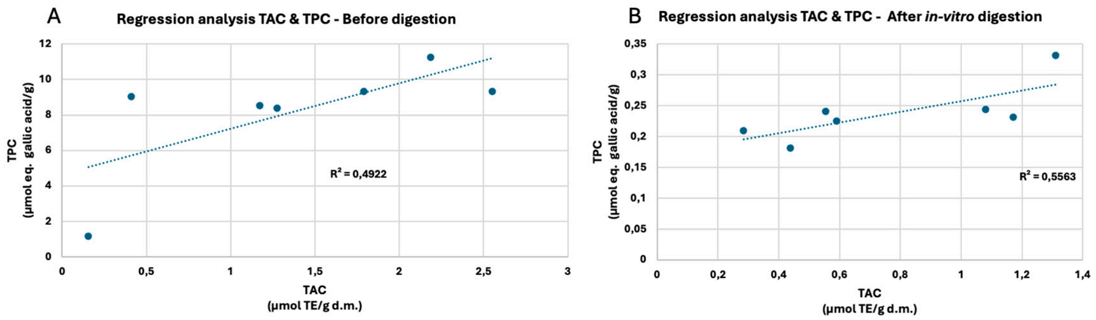

Figure S4. Regression analysis between total antioxidant capacity (TAC) and total polyphenol content (TPC) of chickpea-based fermented beverages (panel A) and the bioaccessible fractions collected after *in vitro* digestion (panel B).
